# 4,6-Dichloro-2-{[(*E*)-(3-{[(*E*)-3,5-dichloro-2-hy­droxy­benzyl­idene]amino}-2,2-dimethyl­prop­yl)imino]­meth­yl}phenol

**DOI:** 10.1107/S1600536811053438

**Published:** 2011-12-17

**Authors:** Hadi Kargar, Reza Kia, Saeideh Abbasian, Muhammad Nawaz Tahir

**Affiliations:** a Department of Chemistry, Payame Noor University, PO BOX 19395-3697 Tehran, I.R. of Iran; b X-ray Crystallography Lab., Plasma Physics Research Center, Science and Research Branch, Islamic Azad University, Tehran, Iran; c Department of Chemistry, Science and Research Branch, Islamic Azad University, Tehran, Iran; d Department of Physics, University of Sargodha, Punjab, Pakistan

## Abstract

In the title compound, C_19_H_18_Cl_4_N_2_O_2_, a potential tetra­dentate Schiff base ligand, the dihedral angle between the two benzene rings is 48.01 (10)°. The configuration about the two C=N bonds is *E* and two intra­molecular O—H⋯N hydrogen bonds make *S*(6) ring motifs. In the crystal, mol­ecules are linked along the *b* axis *via* inter­molecular C—H⋯Cl inter­actions. The crystal structure is further stabilized by an inter­molecular π–π inter­action [centroid–centroid distance = 3.5744 (12) Å].

## Related literature

For standard bond-lengths, see: Allen *et al.* (1987 ▶). For hydrogen bond motifs, see: Bernstein *et al.* (1995 ▶). For related structures, see: Kargar *et al.* (2011 ▶); Kia *et al.* (2010 ▶).
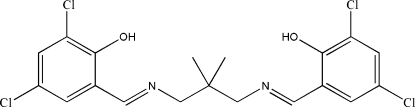


## Experimental

### 

#### Crystal data

C_19_H_18_Cl_4_N_2_O_2_
*M* *_r_* = 448.15
Monoclinic, 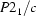

*a* = 16.5265 (5) Å
*b* = 10.3242 (3) Å
*c* = 12.6433 (4) Å
β = 104.796 (1)°
*V* = 2085.70 (11) Å^3^
*Z* = 4
Mo *K*α radiation
μ = 0.58 mm^−1^
*T* = 296 K
0.18 × 0.12 × 0.08 mm

#### Data collection

Bruker SMART APEXII CCD area-detector diffractometer
Absorption correction: multi-scan (*SADABS*; Bruker, 2005 ▶) *T*_min_ = 0.902, *T*_max_ = 0.955
19903 measured reflections
5165 independent reflections
3427 reflections with *I* > 2σ(*I*)
*R*_int_ = 0.027

#### Refinement

*R*[*F* ^2^ > 2σ(*F* ^2^)] = 0.042
*wR*(*F* ^2^) = 0.119
*S* = 1.03
5165 reflections
246 parameters
H-atom parameters constrained
Δρ_max_ = 0.40 e Å^−3^
Δρ_min_ = −0.38 e Å^−3^

### 

Data collection: *APEX2* (Bruker, 2005 ▶); cell refinement: *SAINT* (Bruker, 2005 ▶); data reduction: *SAINT*; program(s) used to solve structure: *SHELXS97* (Sheldrick, 2008 ▶); program(s) used to refine structure: *SHELXL97* (Sheldrick, 2008 ▶); molecular graphics: *SHELXTL* (Sheldrick, 2008 ▶); software used to prepare material for publication: *SHELXTL* and *PLATON* (Spek, 2009 ▶).

## Supplementary Material

Crystal structure: contains datablock(s) global, I. DOI: 10.1107/S1600536811053438/su2348sup1.cif

Structure factors: contains datablock(s) I. DOI: 10.1107/S1600536811053438/su2348Isup2.hkl

Supplementary material file. DOI: 10.1107/S1600536811053438/su2348Isup3.cml

Additional supplementary materials:  crystallographic information; 3D view; checkCIF report

## Figures and Tables

**Table 1:** Hydrogen-bond geometry (Å, °)

*D*—H⋯*A*	*D*—H	H⋯*A*	*D*⋯*A*	*D*—H⋯*A*
O1—H1⋯N1	0.93	1.73	2.553 (2)	147
O2—H2*A*⋯N2	0.90	1.71	2.553 (2)	155
C12—H12*B*⋯Cl1^i^	0.97	2.80	3.749 (2)	167

Symmetry code: (i) 
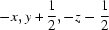
.

**Fig. 1.**
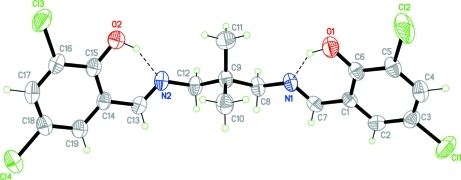
A view of the molecular structure of the title compound, showing 40% probability displacement ellipsoids and the atomic numbering. The dashed lines indicate the intramolecular N-H···O hydrogen bonds (see Table 1 for details).

**Fig. 2.**
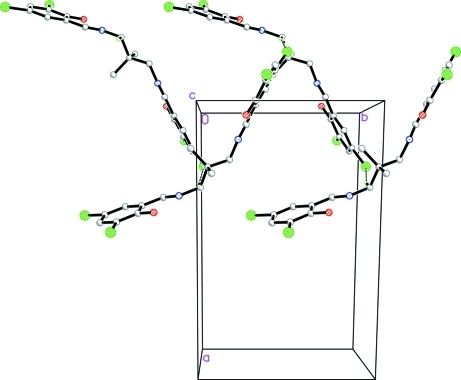
A partial view of the crystal packing of the title compound, viewed down the c-axis, showing the intermolecular C—H···Cl interactions (dashed lines; only the H atoms involved in these interactions are shown].

## supplementary crystallographic information

### Comment

In continuation of our work on Schiff base ligands (Kargar *et al.*, 2011; Kia *et al.*, 2010), we present herein the crystal structure of the title compound.

The title molecule, Fig. 1, is a potential tetradentate Schiff base ligand. The bond lengths (Allen *et al.*, 1987) and angles are within normal ranges and are comparable to those found for similar structures, for example 4,4-Dimethoxy-2,2-[2,2-dimethylpropane-1,3-diylbis(nitrilomethanylylidene)] diphenol (Kargar *et al.*, 2011) and 5,5-Bis(diethylamino)-2,2-[2,2-dimethylpropane-1,3-diylbis(nitrilomethylidyne)] diphenol (Kia *et al.*, 2010). There are two intramolecular O—H···N hydrogen bonds (Table 1) making S(6) ring motifs (Bernstein *et al.*, 1995), and the configuration about both C═ N bonds is E. The two benzene rings, (C1-C6) and (C14-C19), are inclined to one another by 48.01 (10)°.

In the crystal, neighbouring molecules are linked along the *b*-axis direction through intermolecular C—H···Cl interactions (Table 1 and Fig. 2). The crystal structure is further stabilized by an intermolecular π-π interaction involving inversion related molecules [*Cg*1···*Cg*1^i^ = 3.5744 (12)Å; (i) -x, -y, -z; *Cg*1 is the centroid of ring (C1-C6)].

### Experimental

The title compound was synthesized by adding 3,5-dichloro-salicylaldehyde (2 mmol) to a solution of 2,2-dimethyl-1,3-propanediamine (1 mmol) in ethanol (30 ml). The mixture was refluxed with stirring for 30 min. The resultant solution was filtered. Yellow single crystals of the title compound, suitable for *X*-ray analysis, were obtained by recrystallization from ethanol on slow evaporation of the solvent at room temperature over several days.

### Refinement

The OH H-atoms were located in a difference Fourier map and were allowed to ride on the parent O-atom with U_iso_(H) = 1.5U_eq_(O). The C-bound H-atoms were included in calculated positions and treated as riding atoms: C—H = 0.93, 0.96 and 0.97 Å for CH, CH_3_ and CH_2_ H-atoms, respectively, with U_iso_ (H) = k × U_eq_(C), where k = 1.5 for CH_3_ H-atoms, and k = 1.2 for all other H-atoms.

### Crystal data

**Table:**

C_19_H_18_Cl_4_N_2_O_2_	*F*(000) = 920
*M**_r_* = 448.15	*D*_x_ = 1.427 Mg m^−^^3^
Monoclinic, *P*2_1_/*c*	Mo *K*α radiation, λ = 0.71073 Å
Hall symbol: -P 2ybc	Cell parameters from 2540 reflections
*a* = 16.5265 (5) Å	θ = 2.5–27.4°
*b* = 10.3242 (3) Å	µ = 0.58 mm^−^^1^
*c* = 12.6433 (4) Å	*T* = 296 K
β = 104.796 (1)°	Block, yellow
*V* = 2085.70 (11) Å^3^	0.18 × 0.12 × 0.08 mm
*Z* = 4	

### Data collection

**Table:**

Bruker SMART APEXII CCD area-detector diffractometer	5165 independent reflections
Radiation source: fine-focus sealed tube	3427 reflections with *I* > 2σ(*I*)
graphite	*R*_int_ = 0.027
φ and ω scans	θ_max_ = 28.3°, θ_min_ = 2.4°
Absorption correction: multi-scan (*SADABS*; Bruker, 2005)	*h* = −22→22
*T*_min_ = 0.902, *T*_max_ = 0.955	*k* = −13→13
19903 measured reflections	*l* = −16→16

### Refinement

**Table:**

Refinement on *F*^2^	Primary atom site location: structure-invariant direct methods
Least-squares matrix: full	Secondary atom site location: difference Fourier map
*R*[*F*^2^ > 2σ(*F*^2^)] = 0.042	Hydrogen site location: inferred from neighbouring sites
*wR*(*F*^2^) = 0.119	H-atom parameters constrained
*S* = 1.03	*w* = 1/[σ^2^(*F*_o_^2^) + (0.0483*P*)^2^ + 0.5514*P*] where *P* = (*F*_o_^2^ + 2*F*_c_^2^)/3
5165 reflections	(Δ/σ)_max_ = 0.001
246 parameters	Δρ_max_ = 0.40 e Å^−^^3^
0 restraints	Δρ_min_ = −0.38 e Å^−^^3^

### Special details

**Table:**

Geometry. All esds (except the esd in the dihedral angle between two l.s. planes) are estimated using the full covariance matrix. The cell esds are taken into account individually in the estimation of esds in distances, angles and torsion angles; correlations between esds in cell parameters are only used when they are defined by crystal symmetry. An approximate (isotropic) treatment of cell esds is used for estimating esds involving l.s. planes.
Refinement. Refinement of F^2^ against ALL reflections. The weighted R-factor wR and goodness of fit S are based on F^2^, conventional R-factors R are based on F, with F set to zero for negative F^2^. The threshold expression of F^2^ > 2sigma(F^2^) is used only for calculating R-factors(gt) etc. and is not relevant to the choice of reflections for refinement. R-factors based on F^2^ are statistically about twice as large as those based on F, and R- factors based on ALL data will be even larger.

### Fractional atomic coordinates and isotropic or equivalent isotropic displacement parameters (Å^2^)

**Table:**

	*x*	*y*	*z*	*U*_iso_*/*U*_eq_	
Cl1	−0.21903 (4)	−0.02100 (8)	−0.20278 (6)	0.0929 (2)	
Cl2	−0.10588 (6)	0.11487 (9)	0.21661 (6)	0.1149 (4)	
Cl3	0.46259 (4)	0.99049 (7)	0.34212 (5)	0.0831 (2)	
Cl4	0.41065 (4)	1.19135 (6)	−0.06309 (6)	0.0755 (2)	
O1	0.03427 (11)	0.22770 (15)	0.14769 (12)	0.0690 (4)	
H1	0.0791	0.2544	0.1216	0.104*	
O2	0.39245 (10)	0.75500 (14)	0.22723 (12)	0.0621 (4)	
H2A	0.3725	0.6889	0.1818	0.093*	
N1	0.11973 (11)	0.27146 (15)	0.00940 (14)	0.0512 (4)	
N2	0.33256 (10)	0.61812 (16)	0.05705 (15)	0.0540 (4)	
C1	−0.01322 (12)	0.16963 (17)	−0.04155 (15)	0.0436 (4)	
C2	−0.07424 (13)	0.1108 (2)	−0.12407 (17)	0.0529 (5)	
H2	−0.0680	0.1085	−0.1951	0.063*	
C3	−0.14354 (12)	0.0562 (2)	−0.10131 (19)	0.0561 (5)	
C4	−0.15422 (13)	0.05939 (19)	0.0031 (2)	0.0583 (5)	
H4	−0.2016	0.0231	0.0181	0.070*	
C5	−0.09431 (14)	0.11660 (19)	0.08457 (18)	0.0561 (5)	
C6	−0.02244 (13)	0.17359 (17)	0.06566 (16)	0.0488 (5)	
C7	0.06112 (13)	0.22448 (17)	−0.06553 (16)	0.0484 (5)	
H7	0.0657	0.2251	−0.1373	0.058*	
C8	0.19538 (14)	0.32198 (19)	−0.01584 (19)	0.0563 (5)	
H8A	0.1845	0.3363	−0.0940	0.068*	
H8B	0.2399	0.2585	0.0049	0.068*	
C9	0.22377 (12)	0.44956 (17)	0.04474 (16)	0.0478 (4)	
C10	0.15394 (14)	0.5495 (2)	0.0148 (2)	0.0665 (6)	
H10A	0.1729	0.6300	0.0508	0.100*	
H10B	0.1062	0.5192	0.0377	0.100*	
H10C	0.1387	0.5625	−0.0630	0.100*	
C11	0.24805 (17)	0.4257 (3)	0.16823 (19)	0.0722 (6)	
H11A	0.2659	0.5057	0.2057	0.108*	
H11B	0.2929	0.3639	0.1862	0.108*	
H11C	0.2006	0.3927	0.1903	0.108*	
C12	0.30059 (13)	0.49455 (19)	0.00771 (19)	0.0565 (5)	
H12A	0.3442	0.4296	0.0277	0.068*	
H12B	0.2857	0.5031	−0.0713	0.068*	
C13	0.33932 (12)	0.71454 (19)	−0.00290 (18)	0.0517 (5)	
H13	0.3238	0.7055	−0.0786	0.062*	
C14	0.37085 (11)	0.83825 (18)	0.04477 (17)	0.0476 (4)	
C15	0.39724 (12)	0.85146 (19)	0.15984 (17)	0.0496 (5)	
C16	0.42908 (12)	0.9726 (2)	0.20143 (18)	0.0541 (5)	
C17	0.43340 (13)	1.0753 (2)	0.1340 (2)	0.0594 (6)	
H17	0.4546	1.1546	0.1636	0.071*	
C18	0.40590 (13)	1.0599 (2)	0.02195 (19)	0.0552 (5)	
C19	0.37588 (12)	0.9427 (2)	−0.02257 (18)	0.0523 (5)	
H19	0.3588	0.9332	−0.0982	0.063*	

### Atomic displacement parameters (Å^2^)

**Table:**

	*U*^11^	*U*^22^	*U*^33^	*U*^12^	*U*^13^	*U*^23^
Cl1	0.0543 (4)	0.1232 (6)	0.0882 (5)	−0.0138 (4)	−0.0059 (3)	−0.0123 (4)
Cl2	0.1553 (8)	0.1295 (7)	0.0893 (5)	−0.0722 (6)	0.0850 (6)	−0.0384 (5)
Cl3	0.0872 (5)	0.0918 (5)	0.0674 (4)	−0.0259 (4)	0.0148 (3)	−0.0268 (3)
Cl4	0.0754 (4)	0.0543 (3)	0.1042 (5)	0.0008 (3)	0.0363 (4)	0.0125 (3)
O1	0.0863 (11)	0.0739 (10)	0.0535 (9)	−0.0326 (9)	0.0301 (8)	−0.0173 (7)
O2	0.0711 (10)	0.0547 (8)	0.0578 (9)	−0.0074 (7)	0.0115 (8)	−0.0026 (7)
N1	0.0587 (10)	0.0428 (9)	0.0580 (10)	−0.0102 (7)	0.0258 (8)	−0.0038 (7)
N2	0.0503 (9)	0.0469 (9)	0.0671 (11)	−0.0085 (7)	0.0191 (8)	−0.0114 (8)
C1	0.0483 (10)	0.0370 (9)	0.0478 (10)	0.0032 (7)	0.0166 (8)	0.0044 (8)
C2	0.0533 (12)	0.0557 (12)	0.0478 (11)	0.0066 (9)	0.0094 (9)	0.0042 (9)
C3	0.0422 (10)	0.0541 (12)	0.0670 (14)	0.0042 (9)	0.0052 (9)	0.0007 (10)
C4	0.0523 (12)	0.0457 (11)	0.0840 (16)	−0.0002 (9)	0.0305 (11)	−0.0033 (11)
C5	0.0687 (13)	0.0475 (11)	0.0625 (13)	−0.0057 (10)	0.0358 (11)	−0.0058 (10)
C6	0.0617 (12)	0.0361 (9)	0.0540 (11)	−0.0032 (8)	0.0248 (10)	−0.0040 (8)
C7	0.0611 (12)	0.0419 (10)	0.0468 (11)	0.0022 (9)	0.0223 (9)	0.0032 (8)
C8	0.0621 (13)	0.0464 (11)	0.0688 (14)	−0.0103 (9)	0.0319 (11)	−0.0100 (10)
C9	0.0514 (11)	0.0403 (10)	0.0552 (11)	−0.0026 (8)	0.0202 (9)	−0.0067 (8)
C10	0.0596 (13)	0.0503 (12)	0.0909 (17)	0.0019 (10)	0.0214 (12)	−0.0018 (12)
C11	0.0799 (16)	0.0775 (16)	0.0589 (14)	−0.0062 (13)	0.0171 (12)	−0.0038 (12)
C12	0.0573 (12)	0.0485 (11)	0.0693 (14)	−0.0104 (9)	0.0262 (11)	−0.0170 (10)
C13	0.0462 (11)	0.0539 (12)	0.0560 (12)	−0.0055 (9)	0.0148 (9)	−0.0109 (10)
C14	0.0375 (9)	0.0459 (10)	0.0605 (12)	−0.0032 (8)	0.0147 (9)	−0.0068 (9)
C15	0.0398 (10)	0.0483 (11)	0.0615 (13)	−0.0013 (8)	0.0145 (9)	−0.0078 (9)
C16	0.0440 (10)	0.0583 (12)	0.0610 (13)	−0.0061 (9)	0.0150 (9)	−0.0164 (10)
C17	0.0486 (11)	0.0473 (11)	0.0863 (17)	−0.0085 (9)	0.0244 (11)	−0.0151 (11)
C18	0.0461 (11)	0.0479 (11)	0.0763 (15)	0.0008 (9)	0.0244 (10)	0.0006 (10)
C19	0.0432 (10)	0.0545 (11)	0.0613 (13)	−0.0014 (9)	0.0173 (9)	−0.0036 (10)

### Geometric parameters (Å, °)

**Table:**

Cl1—C3	1.738 (2)	C8—H8A	0.9700
Cl2—C5	1.728 (2)	C8—H8B	0.9700
Cl3—C16	1.733 (2)	C9—C10	1.522 (3)
Cl4—C18	1.745 (2)	C9—C11	1.529 (3)
O1—C6	1.330 (2)	C9—C12	1.533 (3)
O1—H1	0.9260	C10—H10A	0.9600
O2—C15	1.326 (2)	C10—H10B	0.9600
O2—H2A	0.8989	C10—H10C	0.9600
N1—C7	1.266 (3)	C11—H11A	0.9600
N1—C8	1.463 (2)	C11—H11B	0.9600
N2—C13	1.273 (3)	C11—H11C	0.9600
N2—C12	1.459 (2)	C12—H12A	0.9700
C1—C2	1.392 (3)	C12—H12B	0.9700
C1—C6	1.403 (3)	C13—C14	1.451 (3)
C1—C7	1.453 (3)	C13—H13	0.9300
C2—C3	1.371 (3)	C14—C19	1.390 (3)
C2—H2	0.9300	C14—C15	1.415 (3)
C3—C4	1.377 (3)	C15—C16	1.406 (3)
C4—C5	1.367 (3)	C16—C17	1.374 (3)
C4—H4	0.9300	C17—C18	1.382 (3)
C5—C6	1.400 (3)	C17—H17	0.9300
C7—H7	0.9300	C18—C19	1.373 (3)
C8—C9	1.536 (3)	C19—H19	0.9300
			
C6—O1—H1	108.4	C9—C10—H10B	109.5
C15—O2—H2A	103.5	H10A—C10—H10B	109.5
C7—N1—C8	120.43 (17)	C9—C10—H10C	109.5
C13—N2—C12	120.43 (19)	H10A—C10—H10C	109.5
C2—C1—C6	120.03 (18)	H10B—C10—H10C	109.5
C2—C1—C7	120.20 (18)	C9—C11—H11A	109.5
C6—C1—C7	119.76 (18)	C9—C11—H11B	109.5
C3—C2—C1	120.40 (19)	H11A—C11—H11B	109.5
C3—C2—H2	119.8	C9—C11—H11C	109.5
C1—C2—H2	119.8	H11A—C11—H11C	109.5
C2—C3—C4	120.7 (2)	H11B—C11—H11C	109.5
C2—C3—Cl1	120.95 (18)	N2—C12—C9	111.85 (16)
C4—C3—Cl1	118.37 (17)	N2—C12—H12A	109.2
C5—C4—C3	119.14 (19)	C9—C12—H12A	109.2
C5—C4—H4	120.4	N2—C12—H12B	109.2
C3—C4—H4	120.4	C9—C12—H12B	109.2
C4—C5—C6	122.4 (2)	H12A—C12—H12B	107.9
C4—C5—Cl2	119.05 (16)	N2—C13—C14	121.18 (19)
C6—C5—Cl2	118.52 (17)	N2—C13—H13	119.4
O1—C6—C5	120.20 (18)	C14—C13—H13	119.4
O1—C6—C1	122.47 (17)	C19—C14—C15	120.27 (18)
C5—C6—C1	117.33 (19)	C19—C14—C13	120.01 (19)
N1—C7—C1	121.28 (18)	C15—C14—C13	119.72 (18)
N1—C7—H7	119.4	O2—C15—C16	120.39 (19)
C1—C7—H7	119.4	O2—C15—C14	122.35 (17)
N1—C8—C9	111.48 (16)	C16—C15—C14	117.26 (19)
N1—C8—H8A	109.3	C17—C16—C15	121.9 (2)
C9—C8—H8A	109.3	C17—C16—Cl3	120.06 (16)
N1—C8—H8B	109.3	C15—C16—Cl3	118.02 (17)
C9—C8—H8B	109.3	C16—C17—C18	119.44 (19)
H8A—C8—H8B	108.0	C16—C17—H17	120.3
C10—C9—C11	110.33 (18)	C18—C17—H17	120.3
C10—C9—C12	110.66 (17)	C19—C18—C17	120.8 (2)
C11—C9—C12	109.80 (18)	C19—C18—Cl4	120.06 (18)
C10—C9—C8	110.00 (18)	C17—C18—Cl4	119.17 (16)
C11—C9—C8	109.78 (17)	C18—C19—C14	120.3 (2)
C12—C9—C8	106.19 (15)	C18—C19—H19	119.8
C9—C10—H10A	109.5	C14—C19—H19	119.8
			
C6—C1—C2—C3	−0.1 (3)	C13—N2—C12—C9	−123.0 (2)
C7—C1—C2—C3	178.47 (18)	C10—C9—C12—N2	58.9 (2)
C1—C2—C3—C4	0.4 (3)	C11—C9—C12—N2	−63.2 (2)
C1—C2—C3—Cl1	−178.43 (15)	C8—C9—C12—N2	178.20 (18)
C2—C3—C4—C5	−0.7 (3)	C12—N2—C13—C14	179.89 (17)
Cl1—C3—C4—C5	178.21 (16)	N2—C13—C14—C19	−178.60 (18)
C3—C4—C5—C6	0.6 (3)	N2—C13—C14—C15	2.1 (3)
C3—C4—C5—Cl2	−177.50 (16)	C19—C14—C15—O2	179.00 (18)
C4—C5—C6—O1	−179.76 (19)	C13—C14—C15—O2	−1.7 (3)
Cl2—C5—C6—O1	−1.6 (3)	C19—C14—C15—C16	−0.8 (3)
C4—C5—C6—C1	−0.3 (3)	C13—C14—C15—C16	178.51 (17)
Cl2—C5—C6—C1	177.81 (15)	O2—C15—C16—C17	−178.70 (18)
C2—C1—C6—O1	179.48 (18)	C14—C15—C16—C17	1.1 (3)
C7—C1—C6—O1	0.9 (3)	O2—C15—C16—Cl3	0.7 (3)
C2—C1—C6—C5	0.1 (3)	C14—C15—C16—Cl3	−179.46 (14)
C7—C1—C6—C5	−178.52 (17)	C15—C16—C17—C18	−0.1 (3)
C8—N1—C7—C1	177.78 (17)	Cl3—C16—C17—C18	−179.55 (16)
C2—C1—C7—N1	−175.71 (18)	C16—C17—C18—C19	−1.2 (3)
C6—C1—C7—N1	2.9 (3)	C16—C17—C18—Cl4	179.39 (15)
C7—N1—C8—C9	137.80 (19)	C17—C18—C19—C14	1.5 (3)
N1—C8—C9—C10	−57.9 (2)	Cl4—C18—C19—C14	−179.09 (15)
N1—C8—C9—C11	63.7 (2)	C15—C14—C19—C18	−0.5 (3)
N1—C8—C9—C12	−177.66 (18)	C13—C14—C19—C18	−179.79 (17)

### Hydrogen-bond geometry (Å, °)

**Table:**

*D*—H···*A*	*D*—H	H···*A*	*D*···*A*	*D*—H···*A*
O1—H1···N1	0.93	1.73	2.553 (2)	147
O2—H2A···N2	0.90	1.71	2.553 (2)	155
C12—H12B···Cl1^i^	0.97	2.80	3.749 (2)	167

Symmetry codes: (i) −*x*, *y*+1/2, −*z*−1/2.
